# Development of Hollow Fiber Membranes Suitable for Outside-In Filtration of Human Blood Plasma

**DOI:** 10.3390/membranes15010016

**Published:** 2025-01-09

**Authors:** David Ramada, Bente Adema, Mohamed Labib, Odyl ter Beek, Dimitrios Stamatialis

**Affiliations:** 1 Advanced Organ Bioengineering and Therapeutics, Faculty of Science and Technology, University of Twente, Zuidhorst 28, Drienerlolaan 5, 7522 NB Enschede, The Netherlands; d.j.loureiroramada@utwente.nl (D.R.); b.adema@utwente.nl (B.A.); o.e.m.terbeek@utwente.nl (O.t.B.); 2 NovaFlux, 1 Wall Street, Princeton, NJ 08540, USA; labib@novaflux.com

**Keywords:** end-stage kidney disease, hemodialysis, dialyzers, outside-in filtration (OIF), blood compatibility, thrombus, hollow fiber membranes

## Abstract

Hemodialysis (HD) is a critical treatment for patients with end-stage kidney disease (ESKD). The effectiveness of conventional dialyzers used there could be compromised during extended use due to limited blood compatibility of synthetic polymeric membranes and sub-optimal dialyzer design. In fact, blood flow in the hollow fiber (HF) membrane could trigger inflammatory responses and thrombus formation, leading to reduced filtration efficiency and limiting therapy duration, a consequence of flowing the patients’ blood through the lumen of each fiber while the dialysate passes along the inter-fiber space (IOF, inside-out filtration). This study investigates the development of HF membranes for “outside-in filtration” (OIF) in HD. In OIF, blood flows through the inter-fiber space while dialysate flows within the fiber lumens, reducing the risk of fiber clogging and potentially extending treatment duration. For the OIF mode, the membrane should have a blood-compatible outer selective layer in contact with the patient’s blood. We develop HFs for OIF via liquid-induced phase separation using PES/PVP (polyethersulphone/polyvinylpyrrolidone) blends. The fibers’ surface morphology (SEM, scanning electron microscopy), chemistry (ATR-FTIR—attenuated total reflection-Fourier transform infrared spectroscopy, XPS—X-ray photoelectron spectroscopy), transport properties, and uremic toxin removal from human plasma are evaluated and compared to commercial HFs. These membranes feature a smooth, hydrophilic outer layer, porous lumen, ultrafiltration coefficient of 13–34 mL m^2^ h^−1^ mmHg^−1^, adequate mechanical properties, low albumin leakage, and toxin removal performance on par with commercial membranes in IOF and OIF. They offer potential for more efficient long-term HD by reducing clogging and systemic anticoagulation needs and enhancing treatment time and toxin clearance.

## 1. Introduction

**Figure 1:**
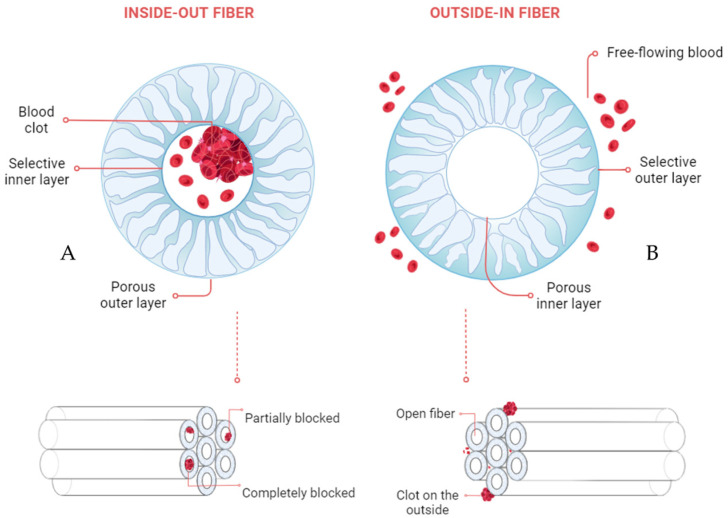
Schematic showing the differences in IOF (inside-out filtration, (**A**)) and OIF (outside-in filtration, (**B**)), detailing each membrane’s morphology.

Patients with end-stage kidney disease (ESKD), whether awaiting a kidney transplant or deemed unsuitable for one, rely on hemodialysis (HD) therapy to remove toxins and excess water from their bodies. This therapy utilizes dialyzers with semi-permeable hollow fiber (HF) membranes to selectively transfer these toxins from the bloodstream into the dialysis fluid (dialysate). Conventional HD is typically performed three times per week in four-hour sessions, but it has limitations in removing middle, large, and protein-bound uremic toxins. Extended or continuous treatments could potentially improve toxin removal [1,2]. To achieve this, dialyzers should be optimized for prolonged use and their membranes should have excellent blood compatibility [3,4,5]. Current dialyzer HFs typically have an asymmetric structure with a thin, selective layer on the lumen side of the fiber and an open porous structure across the rest of the membrane (Figure 1A). The blood flows through the fiber bore, while dialysate flows in the inter-fiber space in the counter-current direction (inside-out filtration, IOF). The thin inner layer determines the sieving properties of the membrane [6,7] while providing a smooth, biocompatible surface that helps mitigate blood interaction [8,9]. In IOF, membrane clogging can occur due to fiber lumen thrombus deposition [10], reducing the functional lifespan of the dialyzers to just 15–45 h for both HD [11] and continuous renal replacement therapy [12], despite advancements in anticoagulation techniques [13,14,15,16]. Some blood oxygenators face the same clotting issues [17] while flowing the blood through the fiber’s lumen, but when these devices are designed to have gas flowing through the fiber lumen and blood in the inter-fiber space, blockages are avoided and a larger surface area is available for gas exchange [18]. Inspired by these blood oxygenators, we develop here HF membranes for outside-in filtration (OIF, Figure 1B). There, the selective layer needs to be on the outside of the fibers since the blood would flow in the inter-fiber space and the dialysate through the fiber bore. Symmetric HFs with selective layers on both sides of the membrane have been reported [19], and while this morphology might reduce the potentially harmful effect of back-filtration of cytokine-induced materials, introducing a second barrier could induce solute accumulation within the membrane wall, decreasing the concentration gradient and the driving force that removes uremic toxins from the blood into the dialysate [20]. Preliminary results of OIF HD have been reported using commercial dialyzers with HFs that have the selective layer in the lumen (IOF) [21], and although they were able to perform HD experiments up to 100 h, clotting still occurred on the outside of the membranes, indicating that a module geometry designed specifically for OIF is also needed. In our group, preliminary results have been presented for mixed matrix membranes (MMM) for OIF studies [22] and showed promise in removing protein-bound uremic toxins from human plasma. Moreover, the first single-layer HF membranes for OIF were also fabricated [23]; however, these membranes need optimization related to transport properties. Therefore, here we develop a new HF for OIF with a high ultrafiltration and low albumin sieving coefficient. Firstly, we focus on optimizing the spinning protocol using different polymer dope compositions to achieve the desired balance between fiber size, morphology, and mechanical and transport properties. We tested three polymer ratios, using a fixed amount of PVP and varying the amounts of PES used. The developed membranes were investigated using SEM, ATR-FTIR, and XPS to assess morphology and surface composition. Subsequent transport measurements are performed to selected OIF HF membranes and compared to commercial FX1000 HF from Fresenius.

## 2. Materials and Methods

### 2.1. Membrane Fabrication

HF membranes were fabricated using liquid-induced phase separation (LIPS). Two polymers—Polyethersulfone (Ultrason E6020 PES, BASF, Ludwigshafen, Germany) and Polyvinylpyrrolidone K90 (BASF K90 PVP, MW = 1–1.5 MDa, BASF, Ludwigshafen, Germany)—were dissolved in ultrapure N-methyl pyrrolidone (NMP) (Sigma-Aldrich, Darmstadt, Germany). A MilliQ purification unit (Merck Millipore, Darmstadt, Germany) provided ultrapure water for all spinning and characterization experiments.

After 2 days of mixing on a roller bench, the polymer solutions were degassed in an ultrasonic bath for 2 h, then transferred to a metal syringe for further degassing over 24 h. Two additional syringes (BD Plastipak Syringe, 60 mL, Stockholm, Sweden) were prepared: one containing the internal coagulant with a 75% NMP and 25% ultrapure water mixture, and an external coagulant with a similar mixture with 17.5% NMP and 82.5% ultrapure water. The three syringes were connected to high-pressure pumps (Fusion 6000, Chemyx, Stafford, TX, USA) with varying flow rates and linked to a spinneret (see Table A1 in Appendix A for dimensions). An air gap of 1.2 or 1.5 cm was set between the spinneret outlet and the coagulation bath surface. Inside the coagulation bath (filled with ultrapure water), three free-spinning wheels guided the nascent fiber to a motorized collection wheel (pulling speed: 9–15 m s^−1^) and then to a collection bucket filled with ultrapure water. The fibers remained in the collection bucket for at least 24 h, with water refreshed periodically to remove residual solvent. The entire fabrication process was conducted at room temperature.

Table 1 summarizes the spinning parameters used for all tested fibers, with three dope compositions varying the PES concentration (8 to 12 wt%) while keeping the PVP concentration constant at 4 wt%.

### 2.2. Membrane Characterization

#### 2.2.1. Scanning Electron Microscopy (SEM)

To assess fiber morphology, each sample was analyzed using scanning electron microscopy (SEM, JEOL JSM-IT 100, Tokyo, Japan). Cross-section samples were prepared by immersing the fibers in liquid nitrogen and cutting them vertically, while inner surface samples were obtained by cutting the fibers horizontally with a microscope and scalpel. The samples were then gold-sputtered (Cressington 108 auto sputter, Cressington Scientific Instruments, Watford, UK) before imaging. All images were taken at specific magnifications: ×150 for the entire cross-section and ×500 for the full thickness of the fiber walls, ×300 for the lumen, and ×1500 for the outer layer. These images were also used to measure outer diameter, inner diameter, and wall thickness using ImageJ software (version 1.54 h), as well as to calculate the surface area of each fiber type.

#### 2.2.2. Attenuated Total Reflectance-Fourier Transform Infrared Spectroscopy (ATR-FTIR)

ATR-FTIR (Spectrum Two, PerkinElmer, Waltham, MA, USA) was used to analyze the surface chemistry of selected fibers. Scans were performed of the outer and inner surfaces of the fibers, at room temperature, in triplicate, and in different zones of the samples for each scan, at a resolution of 4 cm^−1^ for 32 scans and compared to scans of pure PVP and PES. To analyze the inner surface of the fibers, each fiber was longitudinally cut along its length with a scalpel and then flattened with tweezers to expose the inner surface.

#### 2.2.3. X-Ray Photoelectron Spectroscopy (XPS)

XPS analysis of obtained and commercial fibers was performed using a PHI Quantes scanning XPS/HAXPES microprobe (Physical Electronics, Chanhassen, MN, USA) with an Al Kα monochromatic excitation radiation (hυ = 1486.6 eV). Each sample was analyzed 3 times in different regions, and the elemental atomic percentages were averaged.

#### 2.2.4. Mechanical Properties

The evaluation of mechanical properties of the developed fibers was performed with Dynamic Mechanical Analysis (DMA, DMA850, TA Instruments, New Castle, UK), equipped with an 18 N load cell. Fiber samples were air-dried over 24 h and cut to 4 cm, clamped on both ends, and pulled at a constant elongation velocity of 0.5 mm min^−1^, at a constant temperature of 20 °C. Maximum Elongation Before Break (%), Maximum Strength (MPa), and Young’s Modulus (MPa, 0–1% strain used for calculations) were obtained for selected and commercial fibers (FX1000 from Fresenius, Bad Homburg, Germany).

#### 2.2.5. Membrane Performance

##### Clean Water Transport Study

The selected HF membranes were dried at room temperature and used to make mini dialyzers for water transport measurements. Mini-dialyzers were assembled with clear plastic tubing (LDPE, Ø 6 mm) and T-shaped push-in connectors (QST-V0-6, FESTO, Esslinger, Germany), providing 9 cm of usable fiber length. Three fibers were placed in each module, and the ends were sealed with epoxy resin (Griffon Combi Snel-Rapide, Bison International, Goes, The Netherlands). Prior to the water transport experiments, a pre-compacting step of 760 mmHg transmembrane pressure (TMP) was performed for 30 min. Permeated ultrapure water was measured at different TMP values ranging between 375 and 760 mmHg. The ultrafiltration coefficient (K_UF_, mL m^−2^ h^−1^ mmHg^−1^) was calculated as the slope of the linear fit of flux (mL m^−2^ h^−1^) versus TMP (mmHg). For OIF HF, the experiment was performed in OIF mode, and for FX1000 (Fresenius Medical Care, Bad Homburg v. d. Höhe, Germany) in IOF mode.

##### Membrane Sieving Coefficient

The sieving coefficient (SC) of a membrane indicates the membrane transport selectivity. For SC studies, we used five model molecules, all obtained from Sigma-Aldrich (Darmstadt, Germany): creatinine (113 Da, 0.1 mg mL^−1^), myoglobin (17.2 kDa, 0.25 mg mL^−1^), BSA (66 kDa, 1 mg mL^−1^), gamma-globulin (50 kDa, 0.5 mg mL^−1^), and thyroglobulin (660 kDa, 1 mg mL^−1^) in Phosphate-Buffered Saline (PBS, pH 7.4).

**Figure 2:**
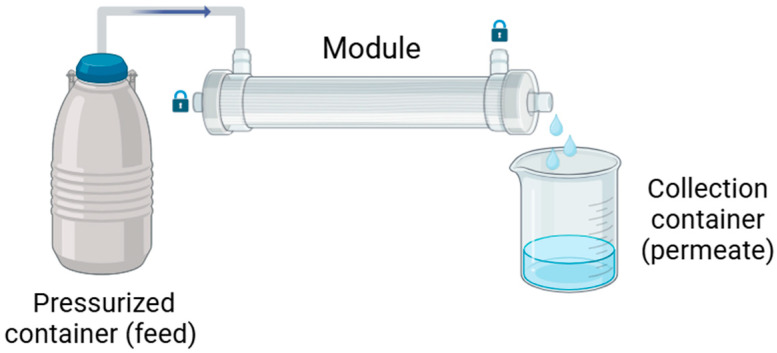
Experimental setup to determine the membrane sieving coefficient (here showing OIF configuration). The pressurized container contains the feed solution that is collected in the collection container (as permeate) after passing through the membrane.

For each solution, three mini dialyzers containing three fibers each (n = 3) were used (Figure 2). After a pre-compacting step of 30 min at 760 mmHg, the transport of model molecules across the HF membranes was measured at 760 mmHg for 30 min. For OIF HF, the experiment was performed in OIF mode, and for FX1000, in IOF mode. The concentration of the tested molecule in the feed and permeate solutions was collected, and their concentrations were measured using UV spectrometry (NanoDrop Technologies, Wilmington, DE, USA). The SC for each molecule was then calculated using Equation (1):

(1) $$SC=\frac{C_{perm}}{C_{feed}}$$

C_perm_ for concentration of model molecules in the permeate and C_feed_ for the concentration in the feed. An SC = 1 indicates that the molecules pass through the membrane unimpeded; SC = 0 indicates that the molecules are completely rejected by the membrane.

##### Uremic Toxin Removal

For toxin removal experiments, human blood plasma and dialysis fluid were prepared in advance. Dialysis fluid was made following an earlier study [24], by dissolving CaCl_2_ (1.5 mM), MgCl_2_ (0.25 mM), NaHCO_3_ (35 mM), NaCl (140 mM), KCl (2 mM) (Sigma-Aldrich, Darmstadt, Germany), and glucose (5.5 mM) (Life Technologies, Bleiswijk, The Netherlands) in ultrapure water (pH 7.4). Human blood plasma obtained by Sanquin (Deventer, The Netherlands) was collected from healthy donors following ethical guidelines.

Creatinine (113 Da), hippuric acid (HA, 179 Da, ~30% albumin-bound), and indoxyl sulfate (IS, 213 Da, ~90% albumin-bound) were all obtained from Sigma-Aldrich (Darmstadt, Germany). These molecules were dissolved in human blood plasma at concentrations similar to those in dialysis patients: creatinine (100 mg L^−1^), HA (110 mg L^−1^), and IS (40 mg L^−1^) [25]. The spiked plasma was shaken at 200 RPM in a 37 °C water bath for 4 h to ensure protein binding for HA and IS.

**Figure 3:**
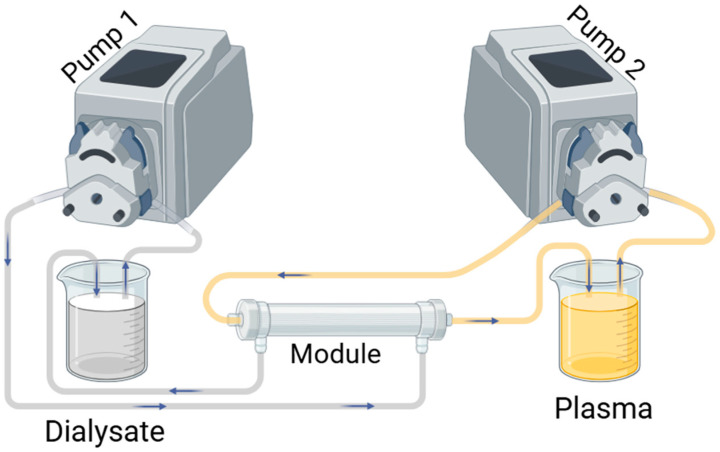
Experimental setup for uremic toxin removal experiments, here showing OIF configuration.

For the transport studies, mini dialyzers (n = 3, 9 cm long) were connected to the Convergence crossflow setup, Figure 3. The total surface area for each of the modules tested is OIF-PES10 #4 (9 fibers): 1.12 × 10^−3^ m^2^, OIF-PES8 #5 (9 fibers): 1.04 × 10^−3^ m^2^ and FX1000 (20 fibers): 1.07 × 10^−3^ m^2^.

Briefly, 50 mL of human plasma and 50 mL of dialysate were recirculated in countercurrent OIF and IOF mode for 4 h. Feed and dialysate flow rates were set at 25 mL min^−1^ and 0.5 mL min^−1^, respectively, to maintain a TMP = 0 mmHg (diffusion experiments). Using these parameters, the flow velocity of feed (plasma) is 18,040 mm s^−1^ and 17,500 mm s^−1^ and dialysate is 15 mm s^−1^ and 13 mm s^−1^ for OIF-PES10 #4 and OIF-PES8 #5, respectively (see Appendix A for detailed calculations).

Samples (0.6 mL) were taken at 0, 30 min, 1 h, 2 h, 3 h, and 4 h. Before analysis, samples were diluted 1:4 with ultrapure water, deproteinized by heat (95 °C, 30 min), and filtered by centrifugation (10 kDa filter, Ultracel-10, Merck Millipore, Darmstadt, Germany) for 15 min at 15,000 RPM). Creatinine concentration was measured using UV–Vis at 230 nm (Nanodrop-1000 spectrophotometer, Fisher Scientific, Waltham, MA, USA). HA and IS concentrations were measured by reverse-phase high-performance liquid chromatography (RP-HPLC, JASCO, Tokyo, Japan) with UV detection at 245 nm and fluorescence at λ_ex_ = 272 nm, λ_em_ = 374 nm. Dialysance for plasma and dialysate fluid (DL_p_ and DL_d_, mL min^−1^ m^−2^) were estimated for all toxins using the following equations [26]:

(2) $${DL}_{p}=\frac{\frac{X_{plasma}}{t.A_{eff}}}{\left(C_{p}-C_{d}\right)}$$

(3) $${DL}_{d}=\frac{\frac{X_{dialysate}}{t.A_{eff}}}{\left(C_{p}-C_{d}\right)}$$

where X_plasma_ (in mg) is the amount of toxins removed from plasma, and X_dialysate_ is the amount of toxins transported to the dialysis fluid (in mg) during a certain amount of time (t). A_eff_ is the effective surface area of the tested module (m^2^; for OIF fibers we consider the outer diameter and the inner diameter for IOF fibers). Finally, C_p_ and C_d_ (in mg mL^−1^) are the concentrations of the toxins in the plasma and dialysis fluid, respectively. If there is no toxin mass imbalance, it is expected that the ratio DL_p_/DL_d_ = 1. However, if there is toxin mass imbalance, the ratio is DL_p_/DL_d_ > 1.

##### Protein Transport

The protein transport/loss from human plasma to the dialysate was assessed during the 4 h experiments. Total protein in plasma and dialysate samples, collected at t = 0 and t = 4 h, was analyzed using UV–Vis at 280 nm (Nanodrop-1000 spectrophotometer, Fisher Scientific, Waltham, MA, USA). Total protein loss from plasma and protein gained in the dialysate were calculated using the following equations:

(4) $$Protein\;loss\;from\;plasma=\frac{P_{t=0}-P_{t=4}}{P_{t=0}}\times 100$$

(5) $$Protein\;gained\;in\;the\;dialysate=\frac{D_{t=4}-D_{t=0}}{D_{t=4}}\times 100$$

where P_t=0_ is the initial protein concentration in plasma, P_t=4_ is the protein concentration in plasma after 4 h, D_t=0_ is the initial protein concentration in dialysate, and D_t=4_ is the final protein concentration in dialysate.

### 2.3. Statistics

All data are presented as a mean ± SD (standard deviation). One-way ANOVA and student’s *t*-test were used as appropriate to compare results between fiber types. All analyses were performed using GraphPad Prism (version 10.3.0). A *p* value of <0.05 was considered significant.

## 3. Results and Discussion

### 3.1. Membrane Fabrication and Morphology

**Figure 4:**
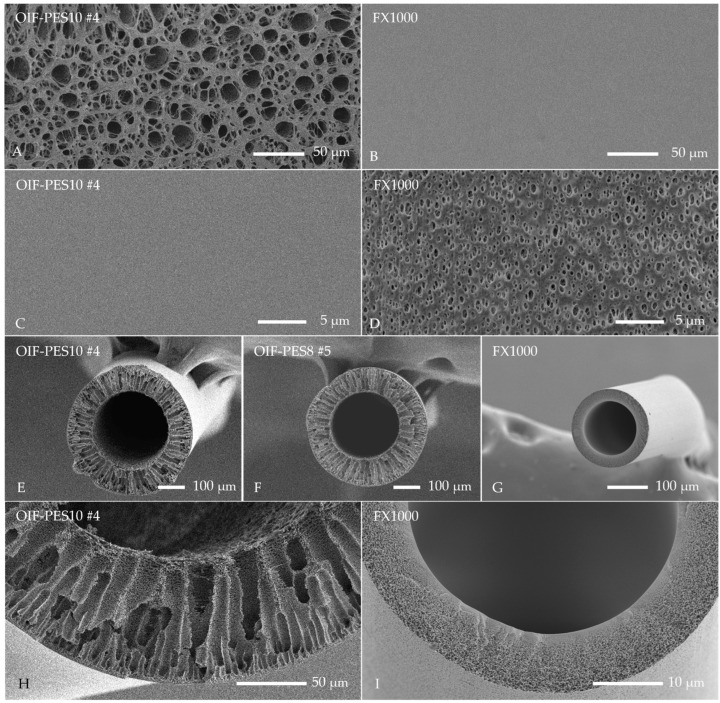
Typical SEM images of OIF-PES10 #4 ((**A**)—lumen, (**C**)—selective outer layer, (**E**)—cross-section, (**H**)—fiber wall), OIF-PES8 #5 ((**F**)—cross-section) and FX1000 ((**B**)—lumen, (**D**)—outer layer, (**G**)—cross-section, (**I**)—fiber wall). Magnifications: cross-section: ×150, fiber wall: ×500, Lumen: ×300 (2B magnification is ×1500 to show surface smoothness), outer layer: ×1500.

**Figure A1:**
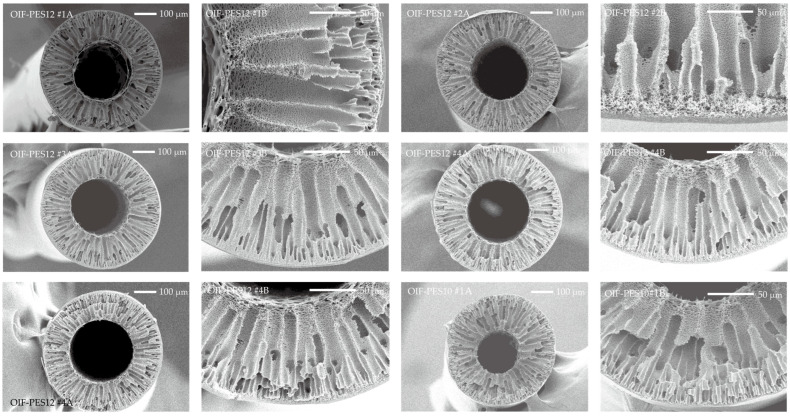

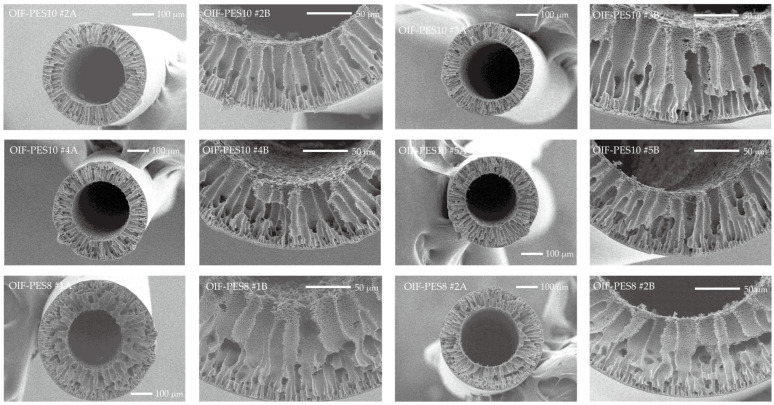

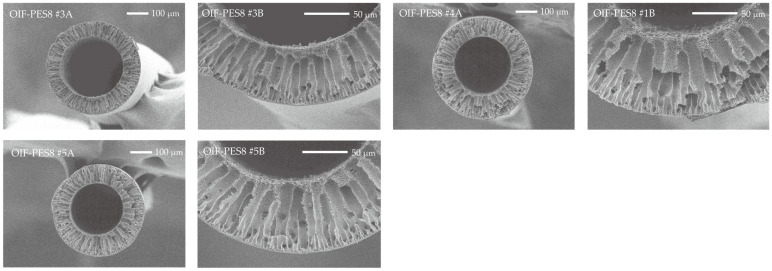
SEM pictures of all the membranes fabricated for this study. Pictures labeled as “A” refer to a cross-section picture taken at ×150 magnification, while B pictures refer to a wall picture taken at ×500 magnification.

Table 1 presents in detail the experimental conditions used for the fabrication of OIF fibers, and Figure 4 presents SEM images of produced fibers compared to commercial Fresenius FX1000 fibers. Additional ReSEM images and characterizations of the developed fibers are presented in Appendix A (Figure A1 and Table A2). The developed HF membranes have an open porous lumen (Figure 4A) and an outer selective layer (Figure 4C). In contrast, the FX1000 HF membranes have an inner selective layer (Figure 4B) and a porous outer layer (Figure 4D). To control fiber dimensions and membrane properties during spinning, we adjusted polymer dope concentration and spinning parameters. The spinneret used (Table A1, Appendix A) was custom designed to produce OIF hollow fibers, incorporating three separate solutions: polymer solution, internal coagulant, and external coagulant. Based on [27,28,29,30], we selected an internal coagulant composition of 75% NMP and 25% water to ensure an open lumen, while an external coagulant mix of 17.5% NMP and 82.5% water was used to produce a thin, smooth outer layer (Figure 4C). Using an NMP/H_2_O mixture allows the thickness and porosity of the lumen and outer layer to be controlled by delaying phase separation [31]. All produced fibers have macrovoids because of low polymer concentration and solvent migration from solvent-rich areas (lumen) to solvent-poor areas (outside of the fiber), in agreement with earlier studies [22]. Furthermore, we recognize that the PES-PVP ratios used in this study (PES-PVP 12–4 wt%, PES-PVP 10–4 wt%, and PES-PVP 8–4 wt%) contribute to the finger-like membrane structure and that may affect the mechanical properties of the membrane, including its elongation at break (see Section 3.3.1).

During spinning, the flowrates of the internal (bore, 0.2 mL min^−1^) and external coagulant (shower, 0.4 mL min^−1^) flow rates, as well as the air gap (1.5 cm), were kept constant (for the formation of a selective outer layer and porous inner layer), but other parameters were adjusted to achieve fibers of different dimensions. A decrease in polymer flow rate (PFR) while maintaining pulling speed (PS) led to a noticeable reduction in fiber diameter, as shown by the 100 µm difference between OIF-PES8 #1 (550 ± 1 µm) and OIF-PES8 #3 (450 ± 1 µm). Increasing PS while keeping PFR constant also reduced fiber diameter, though to a lesser extent, with a 20 µm difference between OIF-PES8 #2 (PS = 12.1 m min^−1^) and OIF-PES8 #4 (PS = 15.3 m min^−1^). Similar trends were observed in the OIF-PES10 fibers (#1 to #3, 90 µm; #2 to #4, 69 µm).

Fiber wall thickness and inner diameter decreased as the outer diameter decreased. Overall, fibers had lower wall thickness and diameter values when PFR was decreased, and PS was increased. The OIF hollow fibers produced here are larger than commercial IOF hollow fibers (Table A2, Appendix A); however, their total surface area in contact with blood or plasma is also larger. Since for IOF, we consider the lumen, and for OIF, the outer surface, for one fiber with 10 cm in length, the effective membrane surface area of the IOF fiber is 5.6 × 10^−5^ m^2^ (for FX1000), while the effective membrane surface area of the OIF fiber of this study is higher (1.2 × 10^−4^ m^2^ for OIF-PES10 #4, 1.2 × 10^−4^ m^2^ for OIF-PES8 #5). Based on the morphology and K_UF_ (data for K_UF_ is discussed in Section 3.3.1), we selected OIF-PES10 #4 and OIF-PES8 #5 for further studies, starting with surface chemistry and transport properties.

### 3.2. Surface Chemistry (ATR-FTIR and XPS)

**Figure 5:**
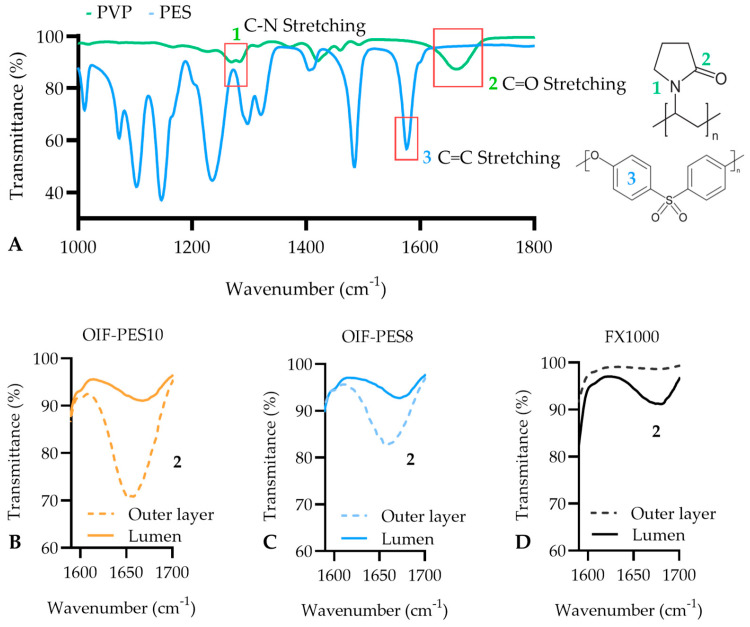
Typical ATR-FTIR of pure PVP in green and pure PES in blue (**A**), highlighting two adsorption bands at wavenumbers 1250–1290 cm^−1^ (C-N stretching vibration) and 1600–1700 cm^−1^ (C=O stretching vibration) for PVP and one for PES at 1485–1580 cm^−1^ (C=C stretching vibration). Below, ATR-FTIR graphs of both the outer layer and lumen side of OIF-PES10 (**B**), OIF-PES8 (**C**), and FX1000 (**D**).

Figure 5 (ATR-FTIR) and Table 2 (XPS) show typical results of surface chemistry analysis of the fibers obtained in this study and of FX1000, for comparison. Figure 5A shows the complete spectra of pure PVP and PES. PVP has two characteristic peaks (C=O stretch at 1600–1700 cm^−1^ and C–N stretch at 1250–1290 cm^−1^) [32]. The C=O stretch peak can be used to assess the presence of PVP on the fibers’ surface as this peak is not present on the PES ATR-FTIR spectra. Based on this, for our OIF fibers, the amount of PVP is higher on the outer fiber surface than on the inner fiber surface (Figure 5B,C); on the contrary, for FX1000, the amount of PVP is higher on the inner fiber surface (Figure 5D). This difference for our OIF HF membranes was expected due to the hydrophilic nature of PVP that causes it to migrate towards areas with higher water content (our outer coagulant has higher water content than the inner coagulant).

Surface atomic composition estimated by XPS measurements was also used to assess the PVP distribution. While carbon and oxygen are present in both PVP and PES, nitrogen is only present in PVP. For the OIF fiber, the results show higher N% in the outer fiber surface compared to the lumen, while for FX1000, N% is very similar to both the outer layer and lumen, consistent with the FTIR results. Significant differences (*p* < 0.05) can be seen for the outer and inner layers of OIF-PES10 #4 and OIF-PES8 #5 (outer and inner layer of FX1000). According to information made available online by Fresenius [33], their membranes are manufactured using mainly polysulfone (Helixone). The presence of nitrogen on the surface of the membrane indicates that PVP is also used in the fabrication of these membranes, which was also discovered by Bowry et al. [34].

In conclusion, SEM, ATR-FTIR, and XPS show that the produced OIF fibers have a selective, outer layer, with a higher amount of PVP there compared to the inner layer. This is particularly important to achieve lower thrombogenicity and minimize membrane fouling.

### 3.3. Membrane Properties

#### 3.3.1. K_UF_, SC, and Mechanical Properties

**Figure 6:**
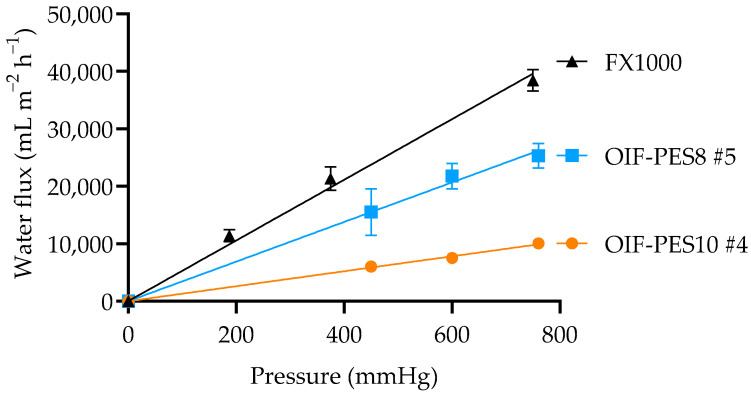
Typical results of CWF vs. TMP for the studied HF membranes (OIF-PES10 #4, OIF-PES8 #5, and FX1000, n = 3 in all cases).

Figure 6 presents the typical results of clean water flux vs. TMP for the tested membranes (OIF-PES10 #4, OIF-PES8 #5, and FX1000).

For all membranes, this graph is linear within the measured pressure range indicating no membrane compaction there. OIF-PES12 fibers prepared using higher polymer concentration have lower K_UF_ than OIF-PES8. OIF-PES10 #4 and OIF-PES8 #5 are in the low-to-medium-flux range, while FX1000 is in the medium-to-high-flux range (52 ± 3 mL m^−2^ h^−1^ mmHg^−1^). Moreover, all membranes were able to withstand pressures up to 750 mmHg, well above the recommended TMP safety limit for HD therapy (of 300 mmHg [35]) during K_UF_ experiments. Handling of the HFs for module fabrication also occurred without any issues.

Table 3 presents the DMA results for the developed OIF HF membranes and FX1000 (results for all fibers in Table A4 in Appendix A). Results show that elastic deformation (Young’s modulus) for OIF-PES10 #4 is the highest, for OIF-PES8 #5 is the lowest, and FX1000 is a value between the two, with significant differences. For max. force before break, FX1000 has the highest value, followed by OIF-PES10 #4 and OIF-PES8 #5. For elongation before break, the values of OIF-PES10 #4 and OIF-PES8 #5 are not significantly different. Nevertheless, the OIF HF membranes were easy to handle and able to withstand pressures up to 760 mmHg.

Table 4 presents the results of the transport studies of various molecules across the membrane at TMP = 760 mmHg and the SC of albumin and molecules with higher molecular weight. Despite differences in dope composition, and experimental parameters used in fabrication and permeability, the SC for all molecules tested is not significantly different between OIF-PES10 #4 and OIF-PES8 #5. Compared to FX1000, which has a different morphology and higher K_UF_, the albumin SC is comparable to our membranes.

The low mechanical properties are due to the low polymer concentration used here, in agreement with other studies [36,37,38,39,40]. In our study, we found that the optimal polymer ratio that offered the highest Young’s modulus values was 10 wt% PES with 4 wt% PVP, which is comparable to the values of the FX1000. If necessary for future upscale studies, we would use higher molecular weight polymers and/or higher polymer concentrations to further improve the mechanical properties of our OIF fibers.

#### 3.3.2. Uremic Toxin Removal Experiments

**Figure 7:**
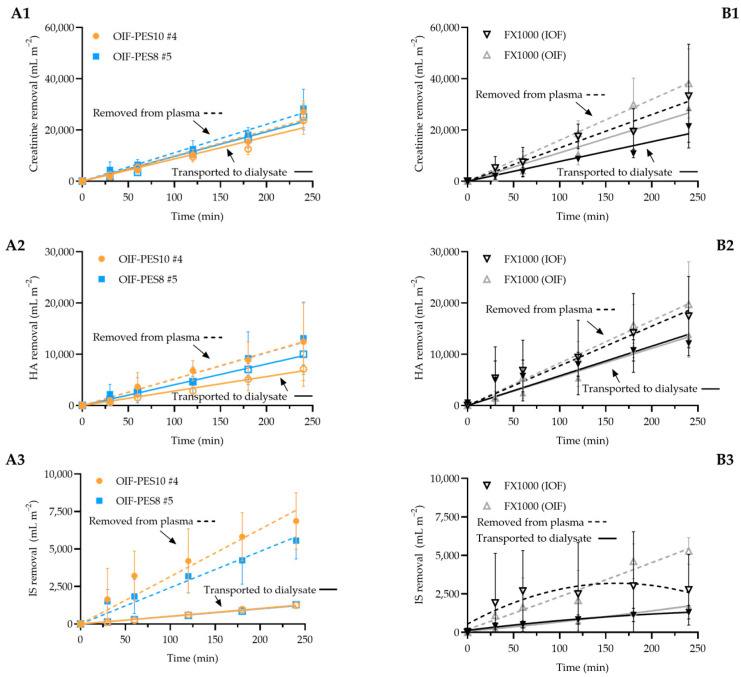
Plasma and dialysate dialysance graphs of our OIF fibers (**A1**–**A3**) and of FX1000 in IOF and OIF modes (**B1**–**B3**). Dotted lunes correspond to toxin removed from plasma and full lines to toxin transported to the dialysate.

Uremic toxin removal from human blood plasma experiments was performed with mini-dialyzers containing OIF-PES10 #4, OIF-PES8 #5, and FX1000 fibers (in IOF and OIF modes), using creatinine, hippuric acid (~30% bound to albumin) and indoxyl sulfate (~90% bound to albumin). Table 5 summarizes the results of 4 h of experiments. Figure 7 presents the kinetics of removal of creatinine, HA, and IS removal (in mL m^−2^) and the slope of these graphs represents the plasma and dialysate dialysance (DLp and DLd, respectively, see Table 5).

For creatinine, total removal was approximately 1100 mg m^−2^, with no statistically significant differences between the tested HFs. Figure 7(A1,B1) show the kinetics of creatinine removed from blood plasma and transported to the dialysate over time. All creatinine removal graphs display linear trends, indicating that a high concentration gradient was sustained across the membrane for the 4 h duration of the experiment. The DL_p_/DL_d_ ratio, ranging between 1 and 2 and consistent across fibers, suggests that transport is predominantly diffusive. In comparison, a prior study [41] using the Fresenius FX1000 Cordiax reported a total creatinine removal of 3533 ± 1526 mg m^−2^ over 4 h in IOF mode. Differences in experimental conditions (1 mL min^−1^ plasma flow rate, 20 mL min^−1^ dialysate flow rate) and the FX1000 Cordiax’s higher K_UF_ (128 mL h^−1^ m^−1^ mmHg^−1^) likely account for the higher removal values observed in that study.

For HA, total removal by all HFs is approximately 900 mg m^−2^, with no statistically significant differences between fibers. Figure 7(A2,B2) show linear trends in HA removal and transport to the dialysate, with DL_p_/DL_d_ ratios ranging from 1 to 2, indicating predominantly diffusive removal, like the findings for creatinine. The lower overall removal of HA compared to creatinine is probably due to its partial binding (~30%) to albumin. A prior study by our group using Baxter Polyflux 2H HFs reported total HA removal from human plasma of around 1500 mg m^−2^ [22]. There, comparable removal was observed in both OIF and IOF modes, consistent with the findings here. Another study [42] using commercial dialyzers (Medica Smartflux HP 120, K_UF_ = 46 mL h^−1^ m^−1^ mmHg^−1^, porcine blood at 150 mL min^−1^, and dialysate at 30 mL min^−1^) also reported similar clearance of HA for both IOF and OIF modes.

For IS, total removal by the studied HFs is between 100 and 250 mg m^−2^, with statistically different results between OIF-PES10 #4 and FX1000 (IOF). Figure 7(A3,B3) illustrate the total amount of IS removed from plasma and transported to the dialysate. For the OIF HFs (Figure 7(A3)), the kinetics of removal are linear over time. For FX1000 (IOF, Figure 7(B3)), the kinetics of IS removal seems to become slower after 3 h, although there is no statistical difference between OIF and IOF per-point removal (at 3 and 4 h). The lower IS removal values compared to creatinine and HA may be attributed to IS’s high (~90%) albumin binding consistent with results of earlier studies [22,41,43]. The DL_p_/DL_d_ ratios for IS are notably higher (3–7) than for the creatinine and HA, suggesting potential interaction/adsorption of IS to the HFs. This is consistent with our earlier findings for IS removal using Polyflux 2H [22]. The same study [22] also showed that the IS removal by FX1000 for both OIF and IOF was similar.

In terms of plasma protein loss (Table 5), the FX1000 HFs exhibit significantly higher protein loss compared to our OIF fibers. Specifically, FX1000 has a total plasma protein loss of approximately 30–40% out of which 15–20% is transported to the dialysate, while our OIF fibers have a total protein loss of 5–10%, with 1–2% transported to the dialysate. These results indicate that a significant part of the PBUT removal from human plasma by FX1000 is due to adsorption combined with protein leakage across the membrane. In an earlier study, we reported similar findings of high removal of HA and IS by FX1000 Cordiax combined with high protein leakage [41]. In contrast, the OIF fibers developed here can achieve high toxin removal mostly via diffusion and have low albumin leakage (Table 4).

It is finally important to note that here, to achieve OIF with TMP = 0, the ratio of flowrate between plasma and dialysate (plasma/dialysate) was adjusted to 25/0.5 mL min^−1^. In other studies for OIF, the plasma and or blood flow rate was also much higher than that of the dialysate, e.g., 10/5 mL min^−1^ [22] or even 100/50 mL min^−1^ [42]. In an OIF configuration, blood/plasma flows around the fibers, creating a less uniform path with slower velocity compared to the flow in the fiber bore. This can lead to insufficient plasma/blood mixing and result in high boundary layer thickness, limiting solute transfer. Conversely, dialysate flows inside the fiber bore, potentially achieving higher velocity leading to reduced mass transfer resistance and improved solute clearance [42]. Therefore, for OIF compared to IOF, it is necessary to utilize a ratio of flowrates between plasma/dialysate higher than one.

## 4. Conclusions

This study presented, for the first time, the development and fabrication of dedicated HF membranes for OIF. The goal of this study was to create membranes specifically designed for OIF mode—with the selective layer on the outside of the fibers and a structure that enhances toxin removal while maintaining essential solute retention and high permeability. The selective outer layer is critical for OIF, as it ensures that direct contact with plasma or blood does not produce any adverse effects due to the activation of coagulation pathways or damage to blood components. However, achieving this design presented significant technical challenges. These included controlling the membrane morphology during fabrication and balancing the trade-off between permeability and selectivity. Our approach successfully overcame these challenges, demonstrating the feasibility of producing OIF-specific fibers. Membrane properties were tailored by optimizing the polymer blends and spinning parameters. The optimal HF membranes had an outer selective layer and a porous lumen, high K_UF_ (13–34 mL m^−2^ h^−1^ mmHg^−1^), adequate mechanical properties, low albumin SC (0.03 ± 0.02), and can successfully remove a range of uremic toxins, including water-soluble and protein-bound.

The development of new OIF fibers is essential for advancing hemodialysis technologies to meet emerging needs, such as prolonged dialysis treatments and the development of wearable or portable dialysis devices. In future studies, we will focus on scaling up the fabrication of these HF membranes and further optimizing the OIF mode of operation. For this, we plan to develop a specially designed dialyzer housing to achieve optimal plasma/blood flow paths in OIF, which could help minimize boundary layers and improve toxin removal. Different plasma/dialysate flow rates will also be tested to ensure proper plasma/blood mixing and the absence of dead-flow zones.

## Figures and Tables

**Table 1:** Experimental parameters used to fabricate OIF hollow fibers. The small arrow points in the direction of what the text is describing.

Membrane Type ↓	Fiber Type	Polymer FR (mL min^−1^)	Bore FR (mL min^−1^)	Shower FR (mL min^−1^)	Air Gap (cm)	PS (m min^−1^)
12 wt% PES, 4 wt% PVP (OIF-PES12)	#1	1.0	0.2	0.4	1.5	9
	#2	1.0	0.2	0.4	1.5	12
	#3	0.8	0.2	0.4	1.5	12
	#4	0.7	0.2	0.4	1.5	14
10 wt% PES, 4 wt% PVP (OIF-PES10)	#1	1.0	0.2	0.4	1.5	12
	#2	0.8	0.2	0.4	1.5	12
	#3	0.6	0.2	0.4	1.5	12
	#4	0.8	0.2	0.4	1.5	15
	#5	0.7	0.2	0.4	1.5	15
8 wt% PES, 4 wt% PVP (OIF-PES8)	#1	1.0	0.2	0.4	1.5	12
	#2	0.8	0.2	0.4	1.5	12
	#3	0.6	0.2	0.4	1.5	12
	#4	0.8	0.2	0.4	1.5	15
	#5	0.7	0.2	0.4	1.5	15

Key: OIF: outside-in filtration; wt%: weight %; FR: flowrate; PS: pulling speed.

**Table 2:** XPS data displaying the elemental surface composition of the outer layer and lumen for OIF-PES10, OIF-PES8, and FX1000. Nitrogen percentage (N%) is highlighted in blue, as PVP is the only polymer containing nitrogen. The small arrow points in the direction of what the text is describing.

Fiber →	OIF-PES10		OIF-PES8		FX1000	
Element ↓	Outer Layer	Lumen	Outer Layer	Lumen	Outer Layer	Lumen
C (%)	73 ± 1	79 ± 2	73 ± 5	73 ± 2	81 ± 1	75 ± 2
N (%)	10 ± 2	4 ± 1	8 ± 4	5 ± 3	4 ± 1	6 ± 2
O (%)	13 ± 1	15 ± 1	16 ± 3	18 ± 3	13 ± 1	17 ± 1
S (%)	3 ± 1	2 ± 0.2	2 ± 0.1	3 ± 0.2	2 ± 0.2	2 ± 1

**Table 3:** Mechanical properties of the studied membranes (n = 3). The small arrow points in the direction of what the text is describing.

Membrane ↓	ID (µm)	OD (µm)	Young’s Modulus (MPa)	Max. Force Before Break (MPa)	Max. Elongation Before Break (%)
OIF-PES10 #4	280 ± 1	440 ± 3	50 ± 1	0.9 ± 0.1	1.9 ± 0.3
OIF-PES8 #5	240 ± 2	409 ± 3	20 ± 4	0.3 ± 0.0	2.1 ± 0.3
FX1000	189 ± 5	266 ± 3	38 ± 4	2.3 ± 0.2	34 ± 7

* *p* < 0.05, ** *p* < 0.01, *** *p* < 0.001, **** *p* < 0.0001. For Young’s modulus, *** OIF-PES10 #4 vs. OIF-PES8 #5, * OIF-PES10 #4 vs. FX1000 and ** OIF-PES8 #4 vs. FX1000. For Fmax, ** OIF-PES10 #4 vs. OIF-PES8 #5, *** OIF-PES10 #4 vs. FX1000, and **** OIF-PES8 #4 vs. FX1000. For Emax, *** OIF-PES10 #4 vs. FX1000 and *** OIF-PES8 #4 vs. FX1000.

**Table 4:** Transport properties of the studied membranes (n = 3) at TMP = 760 mmHg. The small arrow points in the direction of what the text is describing.

	Sieving Coefficient					
Membrane ↓	Creatinine (0.11 KDa)	Myoglobulin (17 KDa)	BSA (66 KDa)	γ-Globulin (150 KDa)	Thyroglobulin (670 KDa)	K_UF_ (mL m^−2^ h^−1^ mmHg^−1^)
OIF-PES10 #4	0.95 ± 0.01	0.89 ± 0.04	0.03 ± 0.02	0.05 ± 0.02	0.06 ± 0.03	13 ± 1
OIF-PES8 #5	0.92 ± 0.08	0.83 ± 0.02	0.03 ± 0.01	0.02 ± 0.01	0.07 ± 0.04	34 ± 3
FX1000	-	-	0.03 ± 0.03	-	-	52 ± 3

**Table 5:** Total toxin removal, dialysance, dialysance ratios, and protein loss in plasma/gained in dialysate for OIF-PES10 #4, OIF-PES8 #5, FX1000 tested in OIF mode and FX1000 tested in IOF mode (n = 3).

	OIF-PES10 #4	OIF-PES8 #5	FX1000 (OIF)	FX1000 (IOF)
K_UF_ (mL m^−2^ h^−1^ mmHg^−1^)	13 ± 1	34 ± 3	52 ± 3	52 ± 3
Total creatinine removal (mg m^−2^) at 4 h	1026 ± 43	1140 ± 187	1196 ± 149	1061 ± 274
DL_p_ creatinine (mL min^−1^ m^−2^)	82 ± 15	108 ± 20	162 ± 39	133 ± 49
DL_d_ creatinine (mL min^−1^ m^−2^)	99 ± 14	96 ± 15	111 ± 34	77 ± 14
DL_p_/ DL_d_ ratio	0.8 ± 0.03	1.1 ± 0.1	1.5 ± 0.1	1.7 ± 0.6
Protein loss in plasma (Cr, %) at 4 h	6 ± 2	11 ± 2	30 ± 12	49 ± 10
Protein gained in dialysate (Cr, %) at 4 h	1 ± 0.2	1 ± 0.02	13 ± 7	17 ± 4
Total HA removal (mg m^−2^) at 4 h	920 ± 282	837 ± 264	933 ± 166	897 ± 261
DL_p_ HA (mL min^−1^ m^−2^)	55 ± 20	48 ± 26	79 ± 14	76 ± 36
DL_d_ HA (mL min^−1^ m^−2^)	27 ± 10	40 ± 6	54 ± 8	56 ± 8
DL_p_/ DL_d_ ratio	2.4 ± 1.2	1.3 ± 0.8	1.5 ± 0.1	1.3 ± 0.5
Total IS removal (mg m^−2^) at 4 h	236 ± 47 *	191 ± 33	166 ± 27	91 ± 54 *
DL_p_ IS (mL min^−1^ m^−2^)	34 ± 11	24 ± 6	22 ± 5	16 ± 14
DL_d_ IS (mL min^−1^ m^−2^)	5 ± 1	5 ± 0.0	7 ± 1	6 ± 2
DL_p_/ DL_d_ ratio	6.5 ± 2.5	4.8 ± 1.2	3.2 ± 0.6	2.6 ± 1.9
Protein Loss in plasma (HA + IS, %) at 4 h	3 ± 2	9 ± 5	25 ± 11	19 ± 5
Protein gained in dialysate (HA + IS, %) at 4 h	2 ± 0.2	2 ± 1	17 ± 4	21 ± 4

* *p* < 0.05.

**Figure A2:**
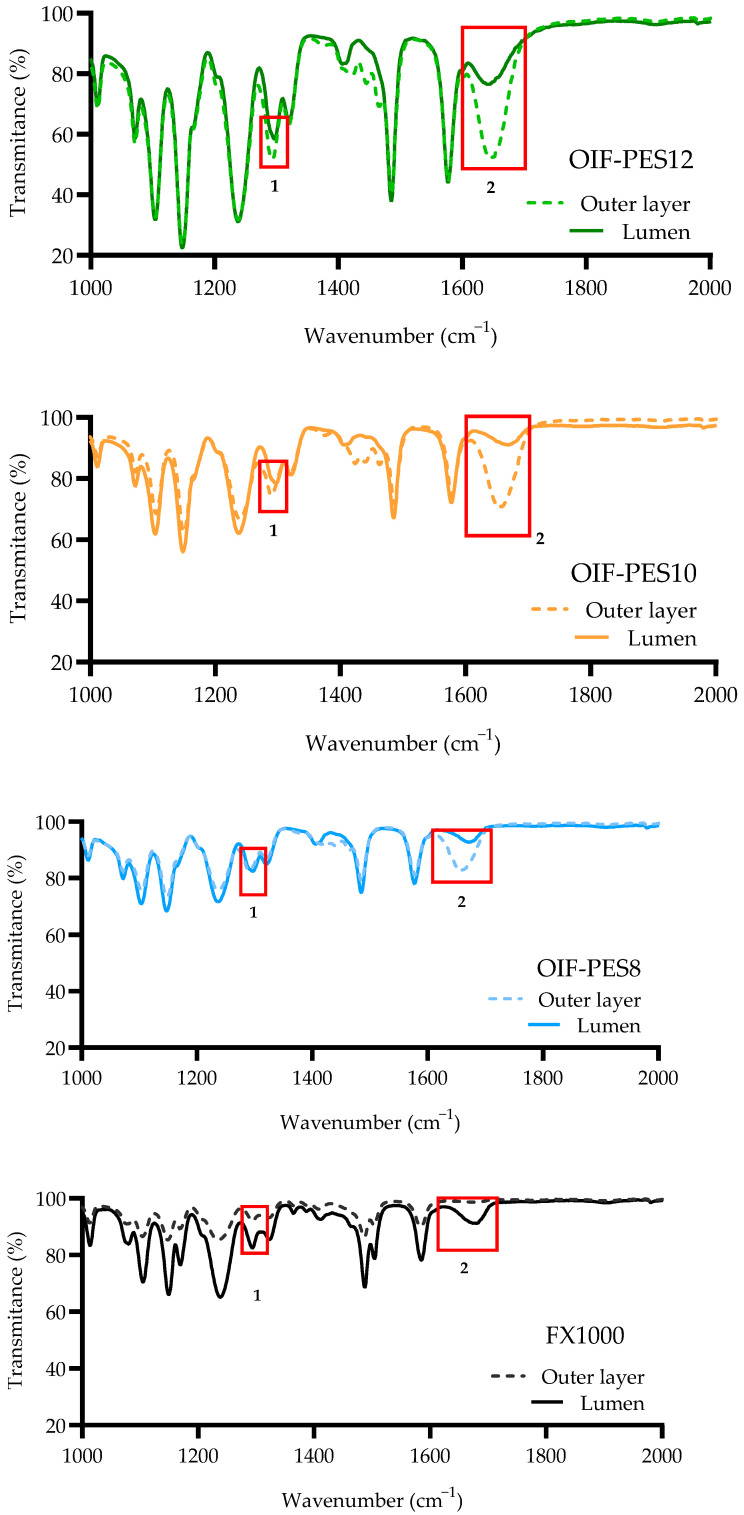
Typical full ATR-FTIR spectra of fiber type OIF-PES12, OIF-PES10, OIF-PES8, and FX1000. Numbers 1 and 2 indicate the 1250–1290 cm^−1^ (C-N stretching vibration) and 1600–1700 cm^−1^ (C=O stretching vibration) of PVP, respectively. Red boxed highlight the previously described wavelength intervals.

## Data Availability

Data are available upon request.

## Appendix A

**Table A1:** Dimensions of the spinneret used.

Inner diameter needle (mm)	0.11 mm
Outer diameter needle (mm)	0.2 mm
Inner diameter first orifice (mm)	0.5 mm
Outer diameter first orifice (mm)	0.8 mm
Inner diameter second orifice (mm)	1 mm

**Table A2:** Dimensions of all obtained fibers (n = 3). Measurements were taken from the SEM pictures, using each picture’s scale as a reference.

Membrane Type	Fiber ID	Outer Diameter (µm)	Inner Diameter (µm)	Wall Thickness (µm)
12 wt% PES, 4 wt% PVP (OIF-PES12)	#1	596 ± 3	310 ± 5	150 ± 3
	#2	576 ± 1	278 ± 4	143 ± 0
	#3	530 ± 4	309 ± 7	119 ± 1
	#4	440 ± 1	300 ± 3	80 ± 2
10 wt% PES, 4 wt% PVP (OIF-PES10)	#1	540 ± 2	290 ± 2	121 ± 9
	#2	509 ± 8	300 ± 2	110 ± 4
	#3	450 ± 1	300 ± 4	85 ± 2
	#4	440 ± 3	280 ± 1	95 ± 1
	#5	409 ± 2	273 ± 4	75 ± 3
8 wt% PES, 4 wt% PVP (OIF-PES8)	#1	550 ± 1	263 ± 1	94 ± 4
	#2	490 ± 5	280 ± 1	95 ± 4
	#3	450 ± 1	296 ± 2	84 ± 1
	#4	470 ± 8	270 ± 1	116 ± 3
	#5	409 ± 3	240 ± 2	101 ± 2
Fresenius	FX1000	266 ± 3	189 ± 5	44 ± 2

**Table A3:** Ultrafiltration coefficient (K_UF_) of obtained and commercial hollow fiber membranes. Three fibers of each type were used per module and tested at different pressure points for 30 min each.

Membrane Type	Fiber Type	Ultrafiltration Coefficient (mL m^−2^ h^−1^ mmHg^−1^)
12 wt% PES, 4 wt% PVP (OIF-PES12)	#1	0.7
	#2	5.9
	#3	7.1
	#4	6.8
10 wt% PES, 4 wt% PVP (OIF-PES10)	#1	13.8
	#2	7.7
	#3	10.9
	#4	13 ± 0.8 (n = 3)
	#5	14.2
8 wt% PES, 4 wt% PVP (OIF-PES8)	#1	23.9
	#2	17.2
	#3	20.6
	#4	25
	#5	34 ± 2.5 (n = 3)
	FX1000	136 [43] 52 ± 3 (this study, n = 3)

**Table A4:** Mechanical properties and outer diameter of the obtained fibers and three commercial fibers (n = 3). Here, we show how our fibers compare to commercial fibers in terms of material stiffness (Young’s modulus, Y_m_), the maximum stress the fibers can withstand before breaking (Max. force before break, F_max_), and the percentage increase in length before the fiber breaks (Max. elongation before break, E_max_).

Membrane Type	Fiber Type	Outer Diameter (µm)	Young’s Modulus (MPa)	Max. Force Before Break (MPa)	Max. Elongation Before Break (%)
12 wt% PES, 4 wt% PVP (OIF-PES12)	#1	596 ± 3	33 ± 3	1.4 ± 0.1	5.2 ± 0.6
	#2	576 ± 1	21 ± 2	1.0 ± 0.1	3.0 ± 0.1
	#3	530 ± 4	28 ± 2	1.5 ± 0.1	3.5 ± 0.4
	#4	440 ± 1	21 ± 1	1.4 ± 0.5	2.6 ± 0.3
10 wt% PES, 4 wt% PVP (OIF-PES10)	#1	540 ± 2	39 ± 2	0.8 ± 0.1	4.0 ± 1.1
	#2	509 ± 8	45 ± 2	0.8 ± 0.0	2.6 ± 0.3
	#3	450 ± 1	40 ± 4	0.8 ± 0.2	2.7 ± 0.8
	#4	440 ± 3	50 ± 1	0.9 ± 0.1	1.9 ± 0.3
	#5	409 ± 2	40 ± 4	0.7 ± 0.1	2.3 ± 0.3
8 wt% PES, 4 wt% PVP (OIF-PES8)	#1	550 ± 1	29 ± 2	0.5 ± 0.0	3.3 ± 0.2
	#2	490 ± 5	33 ± 1	0.5 ± 0.0	1.7 ± 0.1
	#3	450 ± 1	23 ± 3	0.4 ± 0.1	2.3 ± 0.9
	#4	470 ± 8	23 ± 1	0.5 ± 0.1	2.7 ± 1.0
	#5	409 ± 3	20 ± 4	0.3 ± 0.0	2.1 ± 0.3
	FX1000	266 ± 3	38 ± 4	2.3 ± 0.2	34 ± 7

**Table A5:** Data and example calculations of plasma and dialysate flow velocities for OIF-PES10 #4 and OIF-PES8 #5.

Mini-Dialyzer Parameters	OIF-PES10 #4	OIF-PES8 #5
Inner housing diameter (_Dhousing_)	6 mm = 6 × 10^−3^ m	6 mm = 6 × 10^−3^ m
Length of fibers	9 cm	9 cm
Number of fibers (n)	9 fibers	9 fibers
Fiber Dimensions		
Outer diameter (D_outer_)	440 μm = 440 × 10^−6^ m	409 μm = 409 × 10^−6^ m
Inner diameter (D_inner_)	280 μm = 280 × 10^−6^ m	240 μm = 240 × 10^−6^ m
Flow Rates		
Plasma flow rate (Q_plasma_)	25 mL min^−1^ = 4.2 × 10^−7^ m^3^ s^−1^	25 mL min^−1^ = 4.2 × 10^−7^ m^3^ s^−1^
Dialysate flow rate (Q_dialysate_)	0.5 mL min^−1^ = 8.3 × 10^−9^ m^3^ s^−1^	0.5 mL min^−1^ = 8.3 × 10^−9^ m^3^ s^−1^
Plasma flow velocity ($v_{plasma}$)	18,040 mm s^−1^	17,500 mm s^−1^
Dialysate flow velocity ($v_{dialysate}$)	15 mm s^−1^	13 mm s^−1^

Example calculations for OIF-PES10 #4

A. **Cross-Section Areas**
  1. Plasma flow (annular space):
    $$A_{Plasma}=\frac{Pi}{4}({D^{2}}_{housing}-n*{D^{2}}_{outer})$$
    $$A_{Plasma}=\frac{\pi }{4}\left({\left(6\times 10^{-3}\right)}^{2}-9\times ({440\times 10^{-6})}^{2}\right)\approx 2.31\times 10^{-5}\;m^{2}$$
  2. Dialysate flow (lumen of 9 fibers):
    $$A_{dialysate}=n\times 4\pi \times {D^{2}}_{inner}$$
    $$A_{dialysate}=9\times 4\pi \times {\left(280\times 10^{-6}\right)}^{2}\approx 5.54\times 7\;m^{2}$$
B. **Flow velocities**
  1. Plasma velocity:
    $$v_{plasma}=\frac{Q_{plasma}}{A_{plasma}}$$
    $$v_{plasma}=\frac{4.167\times 10^{-7}}{2.31\times 10^{-5}}\approx 18.04\frac{m}{s}=18,040\;mm\;s^{-1}$$
  2. Dialysate velocity:
    $$v_{dialysate}=\frac{Q_{dialysate}}{A_{dialysate}}$$
    $$v_{dialysate}=\frac{8.3\times 10^{-9}}{5.4\times 10^{-7}}\approx 0.015\frac{m}{s}=15\;mm\;s^{-1}$$
